# Techno-economic analysis of a new downstream process for the production of astaxanthin from the microalgae *Haematococcus pluvialis*

**DOI:** 10.1186/s40643-021-00463-6

**Published:** 2021-11-08

**Authors:** Andreas Bauer, Mirjana Minceva

**Affiliations:** Biothermodynamics, TUM School of Life Sciences, Technical University of Munich, Maximus-von-Imhof-Forum 2, 85354 Freising, Germany

**Keywords:** *Haematococcus pluvialis*, Astaxanthin, Techno-economic analysis, Liquid–liquid chromatography, Centrifugal partition extraction, Downstream processing

## Abstract

The biotechnological production of the carotenoid astaxanthin is done with the microalgae *Haematococcus pluvialis* (*H. pluvialis*). Under nutrient deficiency and light stress, *H. pluvialis* accumulates astaxanthin intracellularly and forms a resistant cyst cell wall that impedes direct astaxanthin extraction. Therefore, a complex downstream process is required, including centrifugation, mechanical cell wall disruption, drying, and supercritical extraction of astaxanthin with CO_2_. In this work, an alternative downstream process based on the direct extraction of astaxanthin from the algal broth into ethyl acetate using a centrifugal partition extractor (CPE) was developed. A mechanical cell wall disruption or germination of the cysts was carried out to make astaxanthin accessible to the solvent. Zoospores containing astaxanthin are released when growth conditions are applied to cyst cells, from which astaxanthin can directly be extracted into ethyl acetate. Energy-intensive unit operations such as spray-drying and extraction with supercritical CO_2_ can be replaced by directly extracting astaxanthin into ethyl acetate. Extraction yields of 85% were reached, and 3.5 g of oleoresin could be extracted from 7.85 g homogenised *H. pluvialis* biomass using a CPE unit with 244 mL column volume. A techno-economic analysis was done for a hypothetical *H. pluvialis* production facility with an annual biomass output of 8910 kg. Four downstream scenarios were examined, comparing the novel process of astaxanthin extraction from homogenised cyst cells and germinated zoospores via CPE extraction with the conventional industrial process using in-house or supercritical CO_2_ extraction via an external service provider. After 10 years of operation, the highest net present value (NPV) was determined for the CPE extraction from germinated zoospores.

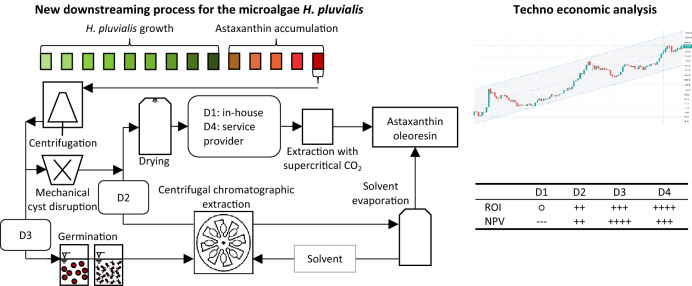

## Introduction

The red carotenoid “astaxanthin” is used as a feed additive for colouring salmon, seafood, and poultry (Shah et al. 2016). It is increasingly used in the cosmetics and dietary supplement industry due to its oxidative characteristics and healthful properties (Li et al. 2020; Astaxanthin Market Size, Share & Trends Analysis Report 2020). Astaxanthin can be chemically synthesised or biotechnologically produced with the microalgae *H. pluvialis* (Nguyen 2013). Due to the increased consumer demand for sustainable ecological products, the market for biotechnologically produced astaxanthin is expected to rise to $148.1 million US (Haematococcus Pluvialis Market 2021).

**Fig. 1:**
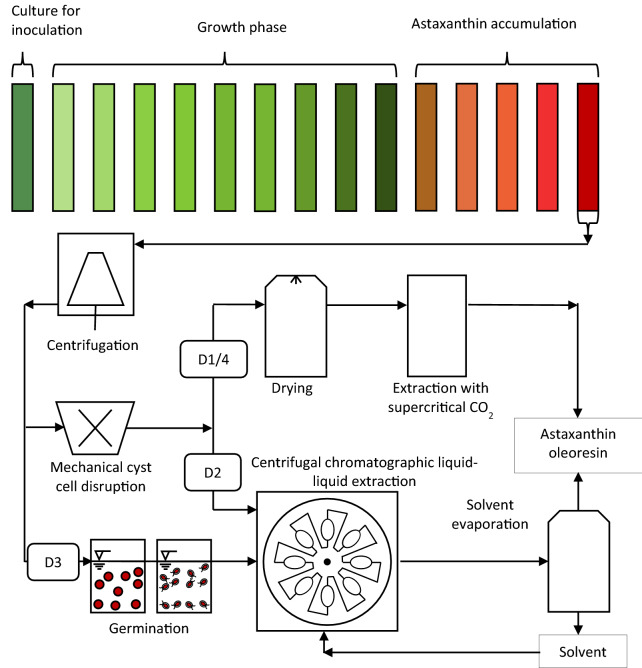
Schematic presentation of the biotechnological production of *H. pluvialis*, including centrifugation, mechanical cyst cell disruption, drying and a supercritical CO_2_ extraction performed in-house (D1) and by an external service provider (D4), as well as liquid–liquid chromatographic extraction of astaxanthin from mechanical disrupted cyst cells (D2) and germinated zoospores (D3) into ethyl acetate, and a subsequent solvent evaporation step

**Fig. 2:**
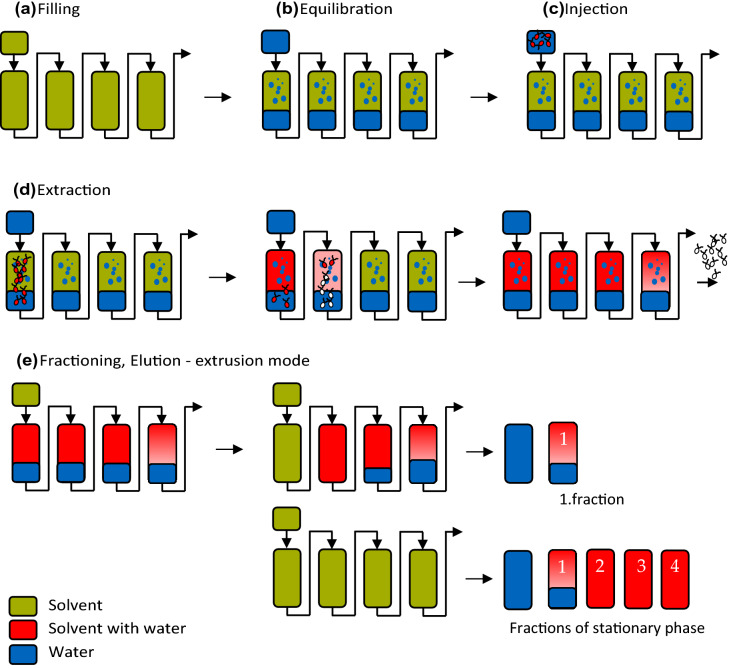
Schematic presentation of the extraction of astaxanthin into a solvent using a CPE column, including **a** filling the column with solvent; **b** equilibration with water; **c** injection of the algal biomass (zoospores or mechanically disrupted cyst cells); **d** extraction of astaxanthin from the aqueous algal broth into the solvent, and **e** fractioning the stationary phase in the elution–extrusion mode

The life cycle of *H. pluvialis* can be divided into mobile and non-mobile phases (Zhang et al. 2017). During favourable growth conditions, the microalgae live mainly as green, flagellated vegetative cells (Fig. 1a). Vegetative cells consist of a cell membrane and an extracellular gelatinous matrix (Hagen et al. 2002). Under stress conditions (nitrate depletion and high light intensity), the vegetative cells become round, expand in cell size, and form immobile aplanospores (Fig. 1b). They accumulate astaxanthin in the cytoplasm of the cell under persistent stress conditions (Fig. 1c) and develop a rigid and resistant cell wall (Fig. 1d) (Hagen et al. 2002; Grünewald et al. 1997). When growth conditions are applied to cyst cells, these form a sporangium and release astaxanthin containing zoospores (Fig. 1e), which only have a thin cell matrix (Fig. 1f). After a specific time, the zoospores become round and form non-motile aplanospores (Fig. 1g). The industrial process is usually performed phototrophically in two steps. In the first stage of the process, the algal biomass is cultivated to reach high cell concentrations under optimal growth conditions, with a sufficient supply of nutrients such as nitrates and phosphates (Nahidian et al. 2018), CO_2_ (Chekanov et al. 2017), and artificial lighting (Katsuda et al. 2004; Xi et al. 2016b). Under nitrate and phosphate deficiency and light stress (Xi et al. 2016a; Sun et al. 2015), the astaxanthin synthesis is initiated in the second step. Astaxanthin accumulation is accompanied by the formation of a resistant cyst cell wall, which impedes direct and efficient astaxanthin extraction. Consequently, a complex downstream process is required, including harvesting the biomass via centrifugation, mechanical cell wall disruption, spray-drying, and the extraction of astaxanthin using supercritical CO_2_ (Panis and Carreon 2016). The current industrial process is shown schematically in Fig. 2, including the conventional downstream process with in-house (D1) supercritical CO_2_ extraction and via an external service provider (D4). Downstream processing in biotechnological processes often represents a bottleneck, showing potential for considerable economic savings (Hatti-Kaul 2000; Minceva and Bauer 2020). Microalgae harvesting may already account for up to 20–30% of the total production costs (Panis and Carreon 2016).

Due to the highly rigid cell wall of the *H. pluvialis* cyst cells, the mechanical cell wall disruption also represents a procedural challenge. Energy-intensive mechanical processes such as bead milling or high-pressure homogenisation are used for industrial cell wall disruption. Up to three-cycle repetitions are needed to achieve sufficient cell wall disruption efficiency using high-pressure homogenisation (Praveenkumar et al. 2020). Drying represents an energy-intensive process step due to the high evaporation enthalpy of water (Δ*h*_vap__._ = 2442 kJ kg^−1^ at 25 °C) (Lide 2005). Spray-drying is commonly used in industry for drying *H. pluvialis* biomass. In this process step, the risk of astaxanthin degradation due to high temperatures or oxidation needs to be considered. The biomass obtained in the drying step needs to show defined densities for it to be processed in the subsequent supercritical CO_2_ extraction. In literature, supercritical CO_2_ extraction of astaxanthin from *H. pluvialis* was intensively studied (Krichnavaruk et al. 2008; Molino et al. 2018). Similar maximum extraction yields of 94% and 92% were reported for supercritical CO_2_ extraction at a pressure of 550 bar and 50 °C (without co-solvent) and 65 °C (with ethanol as co-solvent), respectively (Molino et al. 2018). An increase in the temperature to 80 °C was accompanied by a strong decrease in the yield, which was attributed to the degradation of astaxanthin at these temperatures (Molino et al. 2018). In another study, significantly lower yields of approx. 25% were obtained at a pressure of 400 bar and 70 °C. The yield could be increased to 36% by adding 10 v/v soybean oil as co-solvent (Krichnavaruk et al. 2008). On an industrial scale, up to 1000 bar are used for supercritical CO_2_ extraction of astaxanthin from *H. pluvialis*. Applying pressures ≥ 800 bar and a temperature range from 60 to 80 °C, extraction yields larger than 90% have been reported on an industrial scale (Tippelt 2019).

So far, the biotechnological production of astaxanthin from *H. pluvialis* cannot compete with synthetically produced astaxanthin in terms of production costs (Li et al. 2011). Several studies are presented in the literature to improve the downstream process of biotechnological astaxanthin production (Khoo et al. 2019b). These include alternatives to mechanical cyst disruption using hydrochloric acid or sodium hydroxide, followed by extraction using acetone, where extraction yields of 35% and 30% were reached (Mendes-Pinto et al. 2001). Other alternatives are magnetic-assisted extraction (Zhao et al. 2016) and ultrasound-assisted solvent extraction from dried biomass (Zou et al. 2013). Also, ionic liquids were used to extract astaxanthin from germinated zoospores (Praveenkumar et al. 2015) or dried cyst cell biomass (Liu et al. 2019). Using the CO_2_-based ionic liquid dimethylammonium dimethylcarbamate, extraction yields of 93% were reached for the extraction from dried cyst biomass (Khoo et al. 2021). Using a liquid biphasic floating system composed of 2-propanol and (NH_4_)_2_SO_4_, from 10 mg dried and disrupted *H. pluvialis* biomass dissolved in the salt-rich phase, extraction yields of 95% could be achieved within 15 min (Khoo et al. 2019a). Integrating an ultrasound horn, this process could be further optimised and scaled up to extract 500 mg dried *H. pluvialis* biomass, where yields of 84% could be reached (Khoo et al. 2020). In this study, two novel downstream processes (D2 and D3) were developed to extract astaxanthin from mechanically disrupted cyst cells (D2) or germinated zoospores (D3) directly from the fermentation broth into ethyl acetate using a liquid–liquid chromatographic column. In both cases, the astaxanthin oleoresin could be recovered after evaporation of ethyl acetate. The energy-intensive drying step and extraction with supercritical CO_2_ can be replaced in the novel downstream processes. The new downstream processes were compared with D1 and D4, which present the current industrial downstream process, including mechanical cell wall disruption, homogenisation, drying and extraction with supercritical CO_2_. In scenario D1, the supercritical CO_2_ extraction is performed in-house, in D4 it was considered to be carried out by an external service provider.

The core of the new downstream processes D2 and D3 is the liquid–liquid extraction of astaxanthin from the algal broth into ethyl acetate using a liquid–liquid chromatographic unit. Liquid–liquid chromatography is a solid support-free chromatographic method based on the distribution of solutes between two liquid phases. One of the two liquid phases (ethyl acetate saturated with water in processes D2 and D3) is held stationary in the unit by a centrifugal force. The other phase, the mobile phase (homogenised cyst cells in D2 and germinated zoospores in D3), is pumped through the stationary phase. Dispersion of the mobile phase into the stationary phase occurs, and solutes with lower partition coefficients move faster through the column than those with higher partition coefficients. Depending on the partition coefficients of the solutes, solute separation or extraction can be achieved. If the partition coefficient of a solute is very high, the solute will take a long time to elute from the column. This situation is unfavourable for chromatographic separation, but highly advantageous for extracting astaxanthin from an aqueous algal broth into ethyl acetate. Liquid–liquid chromatographic units exist in hydrodynamic and hydrostatic versions (Ito 2005). In this work, a hydrostatic CPE unit was used. A CPE column is composed of alternately stacked annular plates and annular discs. Chambers are milled into the annular discs, and channels link these chambers. Between two annular discs, an annular plate connects the last chamber of an annular disc with the next through a hole in the annular plate. Annular discs and annular plates are alternately placed on top of each other and mounted on the axis of a centrifuge. A centrifugal force is generated by rotation, and one phase is retained in the chambers (stationary phase, ethyl acetate), while the second phase (mobile phase, algal broth) is pumped through the column from chamber to chamber (Goll et al. 2015). If the mobile phase is the denser phase, this mode is called descending mode; if the mobile phase is the less dense phase, it is called ascending mode. CPE was already used for the extraction of β-carotene from the microalgae *Dunaliella salina* (Marchal et al. 2013) and torularhodin from the yeast *Rhodotorula rubra* (Ungureanu et al. 2013)*.*

In this work, an operating parameter selection of the CPE extraction from mechanically disrupted cysts cells and germinated zoospores was made. A techno-economic study was performed to compare the novel CPE extraction processes from homogenised cyst cells and flagellated zoospores with the industrial supercritical CO_2_ extraction performed in-house or via an external service provider.

## Material and methods

### *H. pluvialis* cyst cell disruption and germination

The biomass for the CPE extraction experiment was provided by the project partner Sea & Sun Technology GmbH, Germany. Either mechanically disrupted cyst cells or germinated zoospores were used for CPE extraction. For germination, cyst cells were harvested, centrifuged at 5500 rpm with a Sigma 3-16KL centrifuge from Sigma GmbH (Germany), and washed with distilled water. A previous publication demonstrated that zoospore release was enhanced under heterotrophic germination conditions compared to photo- or mixotrophic germination (Bauer and Minceva 2021). The highest extraction yield of astaxanthin into ethyl acetate was reached by combining mixotrophic and heterotrophic germination conditions at twice the nitrate concentration of the Bolds modified basal medium (BBM), illumination under mixotrophic conditions until nitrate depletion and subsequent germination under heterotrophic conditions (Bauer and Minceva 2021). Thus, to germinate the cyst cells, these were suspended in BBM with 4 mM glucose and illuminated under mixotrophic conditions for 21 h with a red light (maximum wavelength of 658 nm) and at an intensity of 75 µmol m^−2^ s^−1^ followed by heterotrophic cultivation for 28 h (Bauer and Minceva 2021). Red light was chosen, as higher *H. pluvialis* growth rates compared to fluorescence lamps have been reported in the literature (Katsuda et al. 2004). The germination was done at ambient air without additional CO_2_. CPE extraction was performed 49 h after the start of germination when the maximum zoospore release was achieved. Mechanical cell wall disruption of *H. pluvialis *cyst cells was performed using the APV 1000 high-pressure homogeniser from APV Systems (Denmark) at 750 bar. Mechanical cell wall disruption was carried out in one or three cycles.

### Astaxanthin quantification

The biomass astaxanthin content of the biomass and extracted into ethyl acetate was determined as described in our previous study (Bauer and Minceva 2019). For HPLC analysis, the astaxanthin extract was dissolved in solvent B (methanol, MTBE, water, 8:89:3, v/v) and filtered with a 0.22 µm disposable nylon syringe filter. The astaxanthin quantification was carried out on an HPLC unit (LC-20AB, Shimadzu, Japan), using a YMC carotenoid column (C30, 3 μm, 150 × 4.6 mm, YMC Co., Japan) with a diode array detector (SPD-M20A, Shimadzu, Japan) according to our previous study (Bauer and Minceva 2019). Solvent A (methanol, MTBE, water, 81:15:4, v/v) and solvent B (methanol, MTBE, water, 8:89:3, v/v) were used as the mobile phase. The gradient of the solvent A and B was as follows: 2% solvent B for 11 min, a linear gradient from 2% solvent B to 40% solvent B for 7 min, 40% solvent B for 6.5 min followed by a linear gradient to 100% solvent B for 2.5 min, 100% solvent B for 3 min, a linear gradient to 2% solvent B for 3 min, held for 3 min. The mobile phase flow rate was 1 mL min^−1^ using an injection volume of 10 µL.

### Extraction of astaxanthin from *H. pluvialis* using a centrifugal partition extractor

Extraction experiments were conducted using the centrifugal partition extractor CPC 250 PRO SPECIAL BIO Version (acronym CPE) from Armen Instrument (France), with an experimentally determined column volume of 244 mL (Roehrer and Minceva 2019). The column consists of 12 discs, where each disc has 20 engraved twin-cells resulting in a total of 240 cells. The discs are made of stainless steel and are also coated with polytetrafluoroethylene. The maximum rotational speed achievable was 3000 rpm, with a permitted pressure drop of 100 bar. Two isocratic pumps, model 306 50 C, from Gilson (USA), equipped with an 806 Manometric Module (Gilson, USA), were used to pump the two liquid phases for the CPE extraction experiments.

**Fig. 3:**
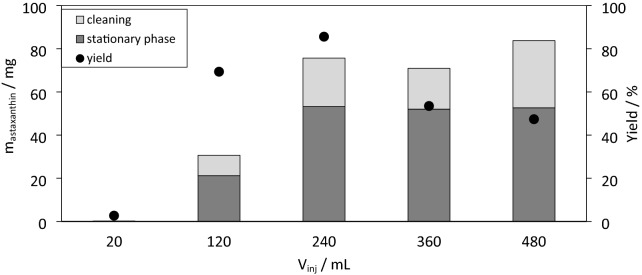
Astaxanthin extracted into the stationary phase and after cleaning the CPE unit with 80 mL acetone, for injection volumes of 20, 120, 240, 360, and 480 mL of homogenised cyst cells with biomass concentrations of 33 g L^−1^, and a flow rate of 40 mL min^−1^ at a rotational speed of 1800 rpm

The process for the extraction of astaxanthin from zoospores or mechanically disrupted cyst cells using a CPE unit is presented schematically in Fig. 3. First, the CPE unit was filled with ethyl acetate (saturated with water) as a stationary phase (see Fig. 3a). The rotation was set to 1800 rpm, and then water (saturated with ethyl acetate) was pumped (see Fig. 3b). Depending on the set flow rate, a specific amount of the stationary phase was displaced from the column.

When the column reaches its hydrodynamic equilibrium, i.e. no more stationary phase leaves the column, the algal broth (zoospores or mechanically disrupted cyst cells) was injected into the column via a 20-mL injection loop (Fig. 3c). The injected biomass concentrations c_DW,injected_ of each experiment are presented in Table 1. After injection, water (saturated with ethyl acetate) was continuously pumped, and astaxanthin was extracted from the aqueous algal broth (zoospores or mechanically disrupted cyst cells) into ethyl acetate (see Fig. 3d). After a predefined time *t*_switch_ (Eq. 1) (Fig. 3d), the solvent-rich phase was pumped into the column (Fig. 3e), causing the displacement of the content from the column. The first fractions collected contained the water-rich phase. After the whole water-rich phase was eluted from the column, the solvent-rich phase—loaded with astaxanthin—was eluted from the column, starting with the least concentrated fraction. The column was cleaned by injecting 80 mL acetone before the next extraction run was performed. Aliquots of the collected stationary phase and acetone used for cleaning were pipetted into 4 mL vials, evaporated using an Alpha 3–4 LSC basic freeze dryer from Martin Christ Gefriertrocknungsanlagen GmbH (Germany) and further processed to analyse the astaxanthin content using HPLC.

**Table 1:** Examined operating conditions of the used CPE column

Cell treatment	*ω*/rpm	*F*/mL min^−1^	*c*_DW,injected_/g L^−1^	*V*_injected_/mL	*m*_DW,injected_/g	*t*_switch_/min
Homogenised	1800	40	72.5	20	1.45	1.9
		20				2.8
		10				4.0
		40	24		0.48	1.9
Homogenised	1800	40	33	20	0.66	1.75
				120	3.96	4.25
				240	7.85	7.25
				360	11.88	10.25
				480	15.84	13.25
Homogenised	1800	40	31.5	240	7.55	7.25
Germinated		40	23	240	5.56	7.25

The switching time, *t*_switch_, was calculated according to Eq. 1, where *V*_MP_ represents the volume of the water-rich phase in the column, *F* the flow rate of the mobile phase, and *V*_inj_ the injection volume of the zoospores or disrupted cyst cells. Equation 1 gives the time theoretically required for the extracted zoospore or disrupted cyst cells to leave the CPE column:

1 $$t_{{{\text{switch}}}} = \frac{{V_{{{\text{MP}}}} + V_{{{\text{inj}}}} }}{F}.$$

The astaxanthin extraction yield *Y* was defined according to Eq. 2, as the sum of the extracted mass of astaxanthin in the stationary phase (solvent) *m*_ATX,SP_ and the amount of astaxanthin recovered after cleaning off the CPE column *m*_ATX,clean_, divided by the amount of astaxanthin in the feed biomass injected, i.e. *m*_ATX,*F*_ (see Fig. 3c):

2 $$Y = \frac{{m_{{{\text{ATX}},{\text{SP}}}} + m_{{{\text{ATX}},{\text{clean}}}} }}{{m_{{{\text{ATX}},F}} }}.$$

Astaxanthin cannot be extracted from cyst cells into ethyl acetate. Hence, *Y* depends on the number of released zoospores or mechanically disrupted cyst cells. Therefore, the yield *Y*_extract_, which considers the actual extractable amount of astaxanthin from the algal broth, was defined (Eq. 3):

3 $$Y_{{{\text{extract}}}} = \frac{Y}{{Y_{{{\text{single-stage}}\;{\text{extraction}}}} }}.$$

The extractable astaxanthin from the feed (zoospores or mechanically disrupted cyst cells) was determined for each extraction experiment by mixing 5 mL algal broth with 5 mL ethyl acetate using a Multi Bio RS-24 shaker from Biosan (Riga, Latvia) for 60 min. The samples were then centrifuged at 5500 rpm for 15 min with a Sigma 3-16KL centrifuge from Sigma GmbH (Germany). The mass of astaxanthin extracted into the solvent divided by the mass of astaxanthin in the feed before extraction defines the yield $Y_{{{\text{single-stage}}\;{\text{extraction}}}} .$

The total extraction time *t*_extraction_ was defined as the sum of filling the column with the stationary phase (*t*_filling_), equilibration time (*t*_equilibration_), the time until the column was empty (*t*_switch_), the time for fractioning the stationary phase (*t*_fractioning_), and the time for cleaning the column (*t*_cleaning_) and is presented in Eq. 4:

4 $$\begin{aligned} t_{{{\text{extraction}}}} & = t_{{{\text{filling}}}} + t_{{{\text{equilibration}}}} + \, t_{{{\text{switch}}}} \\ &\quad+ \, t_{{{\text{fractioning}}}} + \, t_{{{\text{cleaning}}}} . \end{aligned}$$

The conducted CPE experiments are presented in Table 1. The first set of experiments was performed to evaluate the influence of the biomass concentration and mobile flow rate on the extraction performance.

In the second set of experiments, the injection volume of the algal broth was increased from 20 to 480 mL. In the third set of experiments, extraction from germinated zoospores and homogenised cyst cells was done.

## Results and discussion

The objective of this work was to compare four different downstream processing scenarios for the recovery of astaxanthin from *H. pluvialis*. In addition to the existing process, a new process scheme was proposed, performing solvent extraction of astaxanthin from homogenised cyst cells or germinated zoospores using a CPE unit. First, the CPE extraction experiments were conducted using a column with 244 mL volume to evaluate the process performance, followed by a theoretical scale-up of the process to an industrial CPE unit with a 5-L column volume. Subsequently, the mass balances of the upstreaming and downstreaming process of four different scenarios (Fig. 2), supercritical extraction both in-house CO_2_ (D1) and by an external service provider (D4), as well as the extraction of astaxanthin from mechanically disrupted cyst cells (D2) and germinated zoospores (D3) using CPE extraction are discussed. Finally, the total capital investment and total product costs were determined, and an economic analysis was performed.

### Extraction of astaxanthin from *H. pluvialis* using a CPE unit

First, 20 mL of homogenised cyst cells with a concentration of 72.5 g L^−1^ were injected at three different flow rates of the mobile phase: 10, 20, and 40 mL m^−1^. Additionally, at a flow rate of 40 mL min^−1^, a further 20 mL with a biomass concentration of 24 g L^−1^ was injected. The highest extraction yield *Y* of 72% was reached at a flow rate of 40 mL min^−1^ and an injected biomass concentration of 24 g L^−1^. In comparison, a yield of 46% was achieved at a flow rate of 40 mL min^−1^ and an injected biomass concentration of 72.5 g L^−1^. This suggests that a high biomass concentration of 72.5 g L^−1^ compared to 24 g L^−1^ limits the mass transfer of astaxanthin into the solvent due to the increased viscosity at higher biomass concentrations than at lower ones. Extraction yields of 65% and 58% were reached at an injected biomass concentration of 72.5 g L^−1^ and flow rates of the mobile phase of 20 mL min^−1^ and 10 mL min^−1^, respectively. Despite the shorter residence time of the biomass in the CPE unit of 1.8 min at a flow rate of 20 mL min^−1^ compared to 2.0 min at a flow rate of 10 mL min^−1^, the yield was larger at the higher flow rate. Higher contact area between the cells and the solvent result of a better dispersion of the cells in the solvent-rich stationary phase at a higher flow rate at a higher flow rate. The lower yield at a flow rate of 40 mL min^−1^, compared to 20 or 10 mL min^−1^, could be due to the short residence time of 1.4 min of the biomass in the CPE unit at a higher flow rate.

**Fig. 4:**
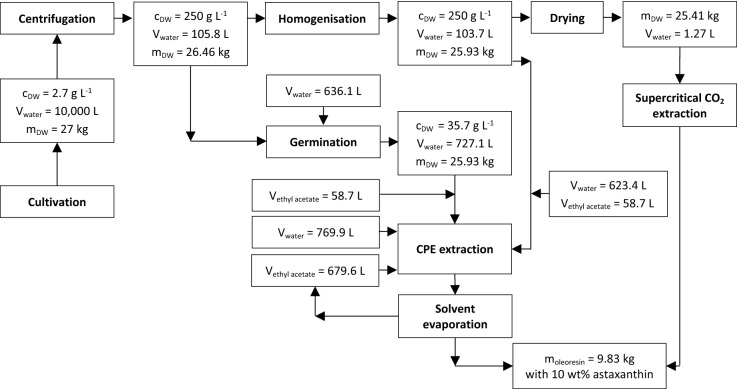
Process flow scheme for the h process steps D1, D2, D3 and D4

The subsequent experiments were performed at a flow rate of 40 mL min^−1^ and low biomass concentrations of 33 g L^−1^ and an astaxanthin content of 1.13 wt%. The injection volume of the homogenised algal broth was gradually increased from 20 to 480 mL. After each experiment, 80 mL of acetone was injected into the CPE column to recover any residues adsorbed onto the CPE column. Figure 4 shows the mass of astaxanthin in the fractions of the stationary phase collected and in the cleaning fraction with acetone. The maximum amount of astaxanthin extracted into the stationary phase is approx. 54 mg for injection volumes of 240 mL to 480 mL. Consequently, the extraction yield calculated using Eq. 2 drops from a maximum of 85% at an injection volume of 240 mL to 48% at 480 mL. This is due to an increasing amount of biomass, leaving the CPE unit non-extracted. After fractionating the stationary phase, significant amounts of astaxanthin could be recovered by injecting 80 mL of acetone. The relatively strong absorption of the carotenoid is probably due to the CPE unit being coated with polytetrafluoroethylene. The injection volume of 240 mL corresponds to an injected biomass of 7.85 g. From the biomass injected, 3.5 g oleoresin with 2.16 wt% astaxanthin was recovered in the stationary phase and cleaning step after the solvent had evaporated.

In the last set of experiments, CPE extraction from homogenised cyst cells was compared to that from flagellated zoospores. Therefore, 240 mL homogenised cyst cells and zoospores with a biomass concentration as reported in Table 1 were injected. Extraction yields of 70% and 80% were reached for the extraction from homogenised cyst cells and zoospores, respectively. In the literature, CPE extraction of β-carotene from living microalgae of *Dunaliella salina* has already been performed. There, extraction yields of 37% and 65% have been reported using decane and ethyl oleate as solvent (Marchal et al. 2013).

### Scale-up of the CPE extractor to an industrial scale

For the scale-up, the results of the experiment with an injection volume of 240 mL homogenised biomass and a concentration of 33 g L^−1^, an astaxanthin content of 1.13 wt% in the biomass, and a flow rate of 40 mL min^−1^ was used. An extraction yield of 85% was reached, and 3.5 g oleoresin with an astaxanthin content of 2.16% was extracted from 7.85 g of biomass with 1.13 wt% astaxanthin. For the scale-up, it was assumed that the astaxanthin content of the biomass of the cyst cells is 5 wt%, and the astaxanthin content in the oleoresin is at around 10 wt%.

Table 2 shows the processing time for one batch extraction, using the experimental data of the experiment with an injection volume of 240 mL and a flow rate of 40 mL min^−1^. The CPE experiment conducted was scaled to a commercially available 5 L CPE from Gilson so that the flow rate resulting in contact time within the column stays the same. As the 5 L CPE unit consists of stainless steel, the column does not need daily cleaning.

**Table 2:** Process step times in CPE columns with a volume of 244 mL and 5 L

	*V*_column_/L	*F*/mL min^−1^	*V*_inj_/mL	*t*_filling_/min	*t*_equilibration_/min	*t*_switch_/min	*t*_fractioning_/min	*t*_cleaning_/min	*t*_extraction_/min
CPE, *V*_inj_ = 240 mL	0.244	40	240	6.1	6.1	7.27	6.1	6.1	31.67
Industrial scenario	5	820	4918	6.1	6.1	7.27	6.1	0	25.57

Table 3 shows the amount of biomass that can be extracted using one industrial 5-L CPE column in 24 h and 330 days. Assuming an annual biomass production of 8910 kg biomass (445.5 kg astaxanthin), three CPE units with a column volume of 5 L would be required, which was used for the downstream processes D2 and D3 in the subsequent techno-economic study.

**Table 3:** Injected amount of biomass, extracted amount of oleoresin and astaxanthin and required number of CPE units in a 24 h and 330 days operation schedule

	*t*_extraction_/min	Injections in 24 h	Biomass injected in		Oleoresin extracted in		Astaxanthin extracted		CPE units
			24 h/kg	330 days/kg	24 h/kg	330 days/kg	24 h/g	330 days/kg	
CPE, *V*_inj_ = 240 mL	31.67	45	0.35	117	0.16	52	15.8	5.2	85.7
Industrial scenario	25.57	56	9.01	2974	4.02	1325	402	132.5	2.9

### Mass balances of the unit steps of the different downstream scenarios

**Fig. 5:**
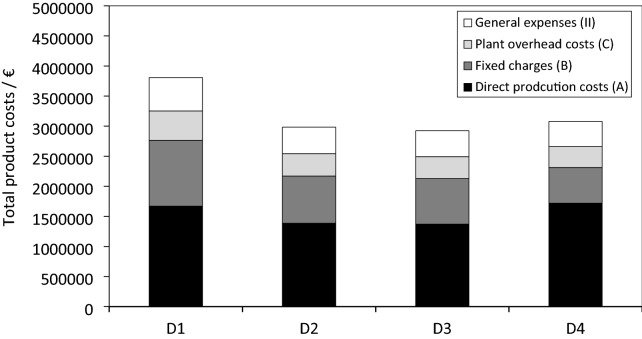
Composition of the total product costs (III), including the direct production costs (A), fixed charges (B), plant overhead costs (C), and general expenses (II) for the four downstream scenarios D1, D2, D3, and D4

The mass flows of each unit operation are presented in Fig. 5. These values were used for calculating the product costs of the process. Subsequently, the assumptions made for the upstreaming process and the unit operations harvesting, cell disruption/germination, spray-drying, CPE extraction, and solvent evaporation are discussed.

#### Upstream processing

A two-stage process was assumed for the upstream process, divided into a 10-day growth phase and a 5-day stress phase for astaxanthin accumulation (Fig. 2). The total photobioreactor volume installed was assumed to be 170 m^3^, as cleaning the harvested reactor (10 m^3^) each day was considered. It was assumed that 10 m^3^ of algal broth with a biomass concentration of 2.7 g L^−1^ and an astaxanthin content of 5 wt% is harvested every day. This means that 8910 kg of biomass can be harvested annually, which is the typical capacity of a small to medium-sized plant (Li et al. 2020). Cultivation on a day–night cycle was assumed so that lighting was required 12 h per day. The installed power was assumed to be 2 W L_algal broth_^−1^. Four rotary vane pumps with a flow of 1200 L h^−1^ each were considered for pumping 10 m^3^ of algal broth. Based on biomass composition of CH_1.83_O_0.48_N_0.11_  (Panis and Carreon 2016) and a CO_2_ conversion rate of 0.75 (Acien et al. 2012), 2.66 kg of CO_2_ was estimated to produce 1 kg algal biomass. CO_2_ was assumed to be dissolved into the algal broth using a CO_2_ sprinkler. The annual nitrogen consumption was calculated based on the chemical composition of the microalgae mentioned, while further nutrients were determined based on their proportion in the Bolts modified basal medium.

#### Harvesting

A disc-stack centrifuge (GEA SEE 10) was considered as a device for harvesting and concentrating the algal broth from an initial 2.7 g L^−1^ (0.27% total suspended solids, TSS) to 250 g L^−1^ (25% TSS). Accordingly, 9894 L can be separated within 4 h, applying a harvesting flow rate of 2500 L h^−1^. The yield for this process step was assumed to be 98%, corresponding to 29.4 kg of cyst cells after the centrifugation step.

#### Cell disruption

Mechanical disruption using a mechanical high-pressure homogeniser (D1, D2, D4) and germination of the cyst cells (D3) was considered for cyst cell disruption. For the mechanical cyst cell disruption, the high-pressure homogeniser GEA Ariete NS3006H was selected. It was considered to operate at a flow rate of 25 L h^−1^, a pressure of 1500 bar, and an operating time of 4.15 h. For cyst cell germination, the approach described in “H. pluvialis cyst cell disruption and germination” section was scaled to a 1000-L reactor. A combination of mixotrophic or heterotrophic germination of cyst cells was considered, where astaxanthin extraction yields of up to 64% could be reached 41 h after the start of germination (Bauer and Minceva 2021). For the scale-up study, two parallel batch photobioreactors with a total volume of 1000 L (727.1 L cultivation volume with a cell concentration of 35.7 g L^−1^) were assumed. Nutrient composition of the BBM with additional 4 mM glucose was considered for germination. As presented in “Extraction of astaxanthin from H. pluvialis using a CPE unit” section, an astaxanthin extraction yield of 80% could be reached for the CPE extraction of germinated zoospores 41 h after the start of germination. This exceeds the reported astaxanthin extraction yield of 64% reported and might be due to differences in the cell status of the microalgae (age, nitrate level, etc.). To the author’s knowledge, the germination of *H. pluvialis* cyst cells is not established on the industrial scale yet, but germination efficiencies were assumed for this study, which would enable a yield of 85% in the subsequent CPE extraction.

The yield of the unit steps of mechanical homogenisation and germination was assumed to be 98%, resulting in 25.93 kg biomass that can be processed in the subsequent unit operations of spray-drying (D1 and D4) or CPE extraction (D2 and D3).

#### Spray-drying

Using the GEA production minor spray dryer, drying with an evaporation rate of 16 L_water_ h^−1^ was considered in this study for drying the algal biomass for subsequent extraction with supercritical CO_2_. The water content of 103.7 L can be reduced to 1.27 L (5 wt%_water_ in the biomass) within around 6.4 h. In this process step, a yield of 98% was considered, which corresponds to 25.41 kg of dry biomass (Panis and Carreon 2016).

#### Extraction with supercritical CO_2_

For in-house supercritical CO_2_ extraction, using a 2 × 40 L (40 L net extractor volume) unit from NATEX Prozesstechnologie GesmbH, with 1000 bar operating pressure, was considered, which can process up to 10 tonnes of biomass within 330 days of annual operation. Applying these pressures, extraction with supercritical CO_2_ can be performed without an additional co-solvent (Tippelt 2019). An annual loss of 1 tonnes of CO_2_ must be considered, according to the manufacturer. For supercritical CO_2_ extraction via an external service provider (D4), it was assumed that the daily produced biomass would be stored at − 20 °C in a cold storage facility until 1000 kg are collected for shipment. As 25.41 kg biomass is collected daily after drying, this corresponds to a 40-day accumulation time.

#### CPE extraction

As presented in “Scale-up of the CPE extractor to an industrial scale” section, three CPE units with a column volume of 5 L are required to process 25.93 kg homogenised cyst cells or zoospores daily. Per batch injection, 0.161 kg algal biomass could be processed within 25.57 min (Table 3). Therefore, 56 batch injections are required daily per CPE unit, corresponding to a daily process time of 22.9 h. For CPE extraction, the solubility of 7.47 v/v ethyl acetate in water and 2.96 v/v water in ethyl acetate must be considered (Stephenson and Stuart 1986). Therefore, the feed must be saturated with ethyl acetate in both scenarios (germinated and homogenised), and the solvent within the CPE column must be saturated with water.

#### Solvent recovery

After the extraction of astaxanthin from the algal broth into ethyl acetate using three 5-L CPE units, 637.8 L of solvent-rich phase, consisting of 618.9 L ethyl acetate and 18.9 L water, must be evaporated daily to receive solvent-free astaxanthin oleoresin. In addition, separation of ethyl acetate from the water-rich phase was considered, although, according to local authorities in Germany, this is not needed for the quantities discharged into wastewater. At atmospheric pressure, the ethyl acetate content in water can be reduced to 0.01 v/v in single-stage evaporation and distillate with approx. 89 v/v ethyl acetate and 11 v/v water can be obtained (Toth 2019). A high-speed evaporator from Ecodyst, with a capacity of 100 L and a maximum evaporation rate of 55 L h^−1^_,_ was considered for solvent evaporation. Given an average evaporation rate of 50 L h^−1^, total evaporation of 637.8 L solvent-rich phase takes 12.8 h, and evaporation of the 119.4 L ethyl acetate from the water-rich phase takes 2.4 h, respectively. Due to the hydrolysis of ethyl acetate to acetic acid and ethanol (Ghobashy et al. 2018), total solvent replacement every 10 days, i.e. 33 times a year, was considered.

### Determination of the total capital investment and total product costs

In the following, the biotechnological production of astaxanthin using the microalgae *H. pluvialis*, and four different downstream processes, supercritical CO_2_ extraction performed in-house (D1), solvent extraction from mechanically disrupted cyst cells (D2) and germinated zoospores (D3), and supercritical CO_2_ extraction performed by an external service provider (D4) are examined about their economic profitability, using the procedure described by Peters and Timmerhaus (1991).

A list of the most important required equipment (TEC) was made for the upstream process and the four downstream scenarios (Turton et al. 2012). This list was used to determine the fixed-capital investments (FCI) and total capital investments (TCI). Finally, the total product costs were calculated as the sum of manufacturing costs and general expenses.

Table 4 lists the most significant equipment costs of the upstream process and the four downstream scenarios. The equipment costs for the upstream process were €965,600, with the costs of the photobioreactors making up about 50% of the upstreaming equipment costs. The equipment costs for the downstream processing are presented in Table 4, where the highest equipment costs for the downstream process, at €1.88 million, were reached for the in-house supercritical CO_2_ extraction (D1), and the lowest cost was calculated for the external supercritical CO_2_ extraction (D4) at €0.58 million. For scenario D4, an additional cooling cell was considered because storage of biomass for around 40 days (up to 1000 kg) was assumed before sending it to the external supercritical CO_2_ extraction service provider. In the conventional downstream processes D1 and D4, the main investment costs are the spray dryer (€450,000), and the additional investment costs of around €1.3 million for the supercritical CO_2_ extractor must be considered for in-house supercritical CO_2_ extraction in D1. The list price of a 1 L CPE column is around €92,000. The purchase price of a 5-L CPC column was estimated from the 1 L CPE column, using the six-tenth-factor rule (Peters and Timmerhaus 1991), resulting in a price of €241,900 per 5-L CPE column. The total direct plant costs are presented in Table 5 and consider the installation costs, instrumentation and control, piping, buildings, yard improvements, service facilities, and land and are then determined by a share of the TEC (Peters and Timmerhaus 1991; Molina Grima et al. 2003). Furthermore, indirect costs, fixed-capital investment (FCI), and the working capital need to be considered to calculate the total capital investment (TCI) (Acien et al. 2012). The course of the TCI correlates directly with the TEC, as it is used to determine the total direct and indirect planned costs (TDIPC), as presented in Table 5.

**Table 4:** Major equipment and total equipment costs (TEC) for the upstream and the four downstream scenarios D1, D2, D3, and D4

List of major equipment	Description	Price/€			
Photobioreactors	170 m^3^				419,900
Light installation	2 W L_algal broth_^−1^				480,000
Pumps for cultivation	68, 1200 L h^−1^ each				27,200
CO_2_ sparkler	17				8500
Medium preparation tank	10 m^3^				30,000
Upstreaming					965,600

		D1	D2	D3	D4
Cooling cell	*A* = 60 m^2^, *k* = 0.25 W m^−2^ K^−1^	–	–	–	7230
Centrifuge	2.5 m^3^ h^−1^	50,000	50,000	50,000	50,000
Homogeniser	50 L h^−1^, 1500 bar	75,000	75,000	–	75,000
Spray dryer	20 L_water_ h^−1^	450,000	–	–	450,000
Supercritical CO_2_ extractor	2 × 40 L, 1000 bar	1,300,000	–	–	–
Reactor and light for germination	2 × 1000 L	–	–	6045	–
CPE units	3 × 5 L columns	–	725,631	725,631	–
Pumps for CPC	1000 mL min^−1^	–	105,000	105,000	–
Rotary evaporator	100 L	–	116,199	116,199	–
Downstream	€	1,875,000	1,071,830	1,002,875	582,230
Total equipment costs (TEC)	€	2,840,600	2,037,430	1,968,475	1,540,600

**Table 5:** List of the total direct plant costs, the indirect plant costs, the fixed-capital investment and the total capital investment of the four downstream processes D1, D2, D3, and D4

		D1	D2	D3	D4
Total equipment costs (TEC)	Share of TEC	2,840,600	2,037,430	1,968,475	1,540,600
Installation costs	0.20	568,120	407,486	393,695	308,120
Instrumentation and control	0.13	369,278	264,866	255,902	200,278
Piping	0.20	568,120	407,486	393,695	308,120
Electrical	0.10	284,060	203,743	196,848	154,060
Buildings	0.23	653,338	468,609	452,749	354,338
Yard improvements	0.12	340,872	244,492	236,217	184,872
Service facilities	0.20	568,120	407,486	393,695	308,120
Land	0.05	142,030	101,872	98,424	77,030
Total direct plant costs	Share of TEC	6,334,538	4,543,469	4,389,700	3,435,538
Engineering supervision	0.30	852,180	611,229	590,543	462,180
Construction expenses	0.10	633,454	454,347	438,970	343,554
Indirect costs		1,485,634	1,065,576	1,029,513	805,734
Total direct and indirect plant costs (TDIPC)	Share of TDIPC	7,820,172	5,609,045	5,419,213	4,241,272
Contractor's fee	0.03	234,605	168,271	162,576	127,238
Contingency	0.07	547,412	392,633	379,345	296,889
Fixed-capital investment (FCI)	Share of TCI	8,602,189	6,169,949	5,961,134	4,665,399
Working capital	0.11	1,063,231	762,606	736,796	576,643
Total capital investment (TCI)		9,665,420	6,932,555	6,697,930	5,242,042

Subsequently, the manufacturing costs (I) were determined: these consist of the direct production costs (A), fixed charges (B), and the plant overhead costs (C) (Peters and Timmerhaus 1991). Finally, the sum of the manufacturing costs (I) and general expenses (II) gives the total product costs (III) and are presented in Table 6. The composition of the manufacturing costs, which are the sum of the direct production costs (A), fixed charges (B), and plant overhead costs (C), will be discussed in the following. The direct production costs include the raw material costs, which were in the range of €30,873 (D4) to €65,253 (D3) and are presented in Table 7 in further detail. The CO_2_ price for cultivation was assumed to be €0.39 per kg (Molina Grima et al. 2003). In total, nutrient costs of €0.50 per kg biomass were calculated, which agrees with reported values of $0.58 US per kg of biomass (Molina Grima et al. 2003). The main water consumption was during the daily harvesting of 10 m^3^ algal broth with water costs of €3.97 m^3^ (VEA: Wasserpreise für Industriekunden bleiben 2016 stabil 2021). The main raw material costs for CPE extraction were solvent costs for ethyl acetate and acetone for cleaning. A loss of 12 tonnes of CO_2_ per year for supercritical CO_2_ extraction results in costs of €4687, assuming a price of €0.39 per kg CO_2_ (Zgheib et al. 2018). For germination, the costs of nutrients, water, and glucose were considered.

**Table 6:** Direct production costs (A), Fixed charges (B) and plant overhead costs (C), manufacturing costs (I, A + B + C), general expenses (II) and total production costs (I + II) of the biotechnological production of astaxanthin from *H. pluvialis* comparing four different downstream scenarios, D1, D2, D3, and D4

	Share of	D1	D2	D3	D4
1. Raw materials		39,347	51,987	58,799	29,096
2. Operating labour	Approx. 15 of (III)	567,188	444,763	436,035	456,408
3. Supervisory/clerical labour	0.12 of (2)	68,063	53,372	52,324	54,769
4. Electricity		503,452	475,599	474,664	462,965
5. Maintenance and repairs	0.04 (of FCI)	344,088	246,798	238,445	186,616
6. Operating supplies	0.1 of (5)	34,409	24,680	23,845	18,662
7. Laboratory charges	0.1 of (2)	56,719	44,476	43,603	45,641
8. Patents and royalties	Approx. 0.015 of (III)	56,828	44,561	43,686	45,728
9. Supercritical CO_2_ extraction via an external service provider	(€50 per kg biomass)				419,301
A. Direct production costs	Sum of (1 to 9)	1,670,092	1,386,235	1,371,401	1,719,185
Lifetime equipment	10 years				
Initial costs for equipment		6,897,829	4,947,491	4,780,049	3,741,039
Salvage value of equipment		689,783	494,749	478,005	374,104
Depreciation equipment per year	10%	620,805	445,274	430,204	336,694
Initial costs for buildings		1,562,330	1,120,587	1,082,661	847,330
Salvage value of buildings		156,233	112,059	108,266	84,733
Depreciation buildings per year	3%	42,183	30,256	29,232	22,878
10. Depreciation total per year		662,988	475,530	459,436	35,9571
11. Local taxes	0.01 of (FCI)	86,022	61,699	59,611	46,654
12. Insurance	0.04 of (FCI)	344,088	246,798	238,445	186,616
B. Fixed charges	Sum of (10 to 12)	1,093,097	784,028	757,493	592,841
C. Plant overhead costs	0.5 of (2, 3, 5)	489,669	372,466	363,402	348,897
I. Manufacturing costs (A + B + C)		3,252,858	2,542,728	2,492,296	2,660,923
14. Administrative costs	0.2 of (2)	113,438	88,953	87,207	91,282
15. Distribution and marketing costs	Approx. 0.05 of (III)	189,067	148,204	145,287	152,276
16. Research and development	0.02 of (IV)	80,009	80,009	80,009	80,009
17. Interest	0.02 of (FCI)	172,044	123,399	119,223	93,308
II. General expenses	Sum of (14 to 17)	554,558	440,565	431,726	416,875
III. Total product cost (I + II)		3,807,416	2,983,294	2,924,022	3,077,798

**Table 7:** Raw material costs of the biotechnological production of *H. pluvialis,* comparing four different downstream scenarios D1, D2, D3, and D4

1. Raw materials costs		D1	D2	D3	D4
Water cultivation		13,101	13,101	13,101	13,101
CO_2_ cultivation		9260	9260	9260	9260
Nutrient cultivation		6736	6736	6736	6736
CO_2_ for extraction		4687	–	–	–
Ethyl acetate (replaced 33 times in 330 days)		–	17,327	17,327	–
Acetone cleaning		5564	5564	5564	–
Water germination		–	–	6.04	–
Nutrient germination		–	–	6736	–
Glucose		–	–	70	–
Total raw material costs	Euro	39,347	51,987	58,799	29,096

The operating labour costs in chemical production facilities are usually between 10 and 20% of the total product cost (III) (Peters and Timmerhaus 1991). In this work, a figure of 15% was assumed. Based on the operating labour costs, the supervisory labour costs and laboratory charges can be estimated (Table 6). The expenses for maintenance and repairs, patents and royalties were calculated as shown in Table 6. Electricity consumption and costs are presented in more detail in Table 8.

**Table 8:** Annual electricity costs of the biotechnological production of *H. pluvialis*, comparing four different downstream scenarios, D1, D2, D3, and D4

4. Electricity costs		D1	D2	D3	D4
Electricity price per kW h^−1^	€0.18				
Pumps and mixing	MWh a^−1^	592	592	592	592
Light	MWh a^−1^	1188	1188	1188	1188
Temperature control	MWh a^−1^	660	660	660	660
Control and sensors	MWh a^−1^	65.9	65.9	65.9	65.9
Upstream	MWh a^−1^	2506.3	2506.3	2506.3	2506.3
Cooling cell (*k* = 0.5 W m^−2^ K^−1^) *A* = 60 m^2^ Δ*T* = 40 K	MWh a^−1^	–	–	–	4.75
Centrifugation	MWh a^−1^	5.28	5.28	5.28	5.28
Homogenisation	MWh a^−1^	7.53	7.53	–	7.53
Spray-drying	MWh a^−1^	48.2	–	–	48.2
Germination	MWh a^−1^	–	–	2.34	–
CPE extraction	MWh a^−1^	–	56.6	56.6	–
Solvent evaporation	MWh a^−1^	–	66.5	66.5	–
Supercritical CO_2_ extraction	MWh a^−1^	229.7	–	–	–
Downstream	MWh a^−1^	290.7	135.9	130.7	65.7
Total electricity	MWh a^−1^	2797.0	2642.2	2637.0	2572.0
Total electricity costs	Euro	503,452	475,599	474,664	462,965

An electricity price of €0.18 kWh^−1^ was assumed for Germany (Industriestrom: Vergleich für Unternehmen 2021).

The total electricity costs for upstreaming are 2506.3 MWh a^−1^ (Table 8) to produce 8.9 tonnes of biomass. However, the exact power consumption varies greatly, depending on the type of cultivation, closed vs. open systems, climatic zone and temperature of the cultivation location, additional lighting (Panis and Carreon 2016; Acien et al. 2012). In a model calculation for the annual production of 18.3 tonnes and 6.15 tonnes of wet *H pluvialis *biomass in Livadeia (Greece) and Amsterdam (Netherlands), the energy consumption of 444.8 MWh a^−1^ and 291 MWh a^−1^ were considered for the upstreaming (Panis and Carreon 2016). In that work, the cultivation was carried out without artificial light. The green phase was conducted in closed photobioreactors, and astaxanthin accumulation was performed in open ponds. In a hypothetical industrial scenario, based on real production data, for the annual production of 17 tonnes of *P. tricornutum* in Germany using artificial light and a total cultivation volume of 315 m^3^, the electricity consumption of 92,916.8 MWh a^−1^ was determined for upstreaming (Derwenskus et al. 2020). This study considered the power consumption of 1100 W for mixing and circulation of the biomass results per rotary vane pump. Due to the lack of real production data, an artificial light installation of 2 W L_algal broth_^−1^ and lighting at 12 h intervals were assumed, resulting in annual electricity consumption of 1188 MWh a^−1^. For temperature control, values of 6.25–25 kWh m^−3^ were reported for *H. pluvialis* cultivation in Shenzhen, China (Li et al. 2011). Therefore, 12.5 kWh m^−3^ was assumed as the energy consumption for temperature control in this study. Power consumption for control and sensors was taken from literature and was adjusted to the cultivation volume of 160 m^3^ in this study (Derwenskus et al. 2020).

In the downstream process of *H. pluvialis*, the highest energy consumption was calculated for in-house supercritical CO_2_ extraction (D1) with 290.7 MWh a^−1^, while reduced electricity consumption levels of 135.9 MWh a^−1^ and 130.7 MWh a^−1^ were calculated for solvent extraction from homogenised cyst cells (D2) and flagellated zoospores (D3), respectively. The lowest electricity consumption, of 65.7 MWh a^−1^, was calculated for the process with external supercritical CO_2_ extraction (D4). The energy consumption for centrifugation was 5.28 MWh a^−1^ in all four scenarios, corresponding to 1.6 kWh m_algal broth_^−3^. The installed power of the disc-stack centrifuge was 4 kW, with a daily operating time of 4 h and a harvesting volume of 10 m^3^. Values of 1–1.4 kWh m_algal broth_^−3^ have been reported for centrifugation in the literature (Panis and Carreon 2016; Milledge 2013). The electricity consumption levels for mechanical cell wall disruption by homogenisation (D1, D2, and D4) and germination (D3) were 6.97 MWh a^−1^ and 5.72 MWh a^−1^, respectively. The costs for homogenisation were determined from the installed power of 5.5 kW of the used homogeniser and a daily operating time of 3.84 h.

Energy costs of 5.72 MWh a^−1^ were calculated for germination, using the data from the upstreaming scenario, and these were transferred to 2 × 1000 L photobioreactors. Lighting for 21 h per germination process was assumed (“H. pluvialis cyst cell disruption and germination” section).

The energy costs for spray-drying were calculated to be 48.2 MWh a^−1^. To determine these costs, the daily amount of water to be evaporated (102.45 kg) was multiplied by the evaporation enthalpy of water (Δ*h*_evaporation_ = 2442.3 kJ kg^−1^ at 25 °C (Lide 2005)) and a factor of 2.1. This factor was suggested by the manufacturer and is in good agreement with efficiencies of 40% and 55% reported in the literature for spray dryers without and with heat recovery, respectively (Kemp 2012) This corresponds to the energy consumption of 5.13 MJ kg_water_^−1^ and fits well to the values of 5 MJ kg_water_^−1^ reported in the literature for this unit operation (Thomassen et al. 2016). For the electricity costs for the CPE extraction (scenario D2 and D3), 2.5 kW needs to be considered according to the manufacturer Gilson (USA). The daily process time per CPE system was 22.9 h (“H. pluvialis cyst cell disruption and germination” section).

The selected evaporator for solvent evaporation has an installed power of 13.3 kW, resulting in annual energy consumption of 66.5 MWh a^−1^, when a daily operating time of 15.2 h is considered (“Solvent recovery” section). The power consumption of 29 kW h^−1^ for the extraction with supercritical CO_2_ was provided by the manufacturer, resulting in annual energy consumption of around 229.7 MWh a^−1^.

Concerning electricity costs, it could be shown that the extraction of astaxanthin from *H. pluvialis* using CPE extraction (D2 and D3) saves electricity costs compared to in-house extraction with supercritical CO_2_ (D1) since energy-intensive unit operations such as spray-drying and supercritical CO_2_ extraction can be replaced. Slightly lower electricity consumption for germination (2.34 MWh a^−1^) can be expected compared to high-pressure homogenisation (7.53 MWh a^−1^). In scenario D4, where supercritical CO_2_ extraction via an external service provider is done, the operation of a cooling cell (*T* = − 20 °C) was considered for storage of the harvested biomass up to 1000 kg before shipment. Therefore, additional energy consumption of 4.75 MWh a^−1^ was considered.

The highest direct production costs (A) were found to be €1.72 million and €1.67 million for external (D4) and in-house (D1) supercritical CO_2_ extraction. In comparison, production costs of around €1.4 million can be expected for the solvent extraction of astaxanthin from homogenised cyst cells (D2) and germinated zoospores (D3). For the supercritical CO_2_ extraction via an external service provider (D4), lower costs for electricity, raw materials, and repairs are outweighed by the payments for the external service provider (€419,301, €50 kg_DW_^−1^). To determine the manufacturing costs (I), in addition to the direct production costs (A), the fixed charges (B) and plant overhead costs (C) need to be determined (Table 6). The fixed charges are the sum of depreciation for equipment and buildings, local taxes, and insurances (Peters and Timmerhaus 1991). A linear depreciation period of 10 years and a residual value of 10% of the original value were assumed for the equipment costs (Turton et al. 2012). The buildings were depreciated by 3% annually (Peters and Timmerhaus 1991). Local tax and insurance costs were considered 1% and 4% of the FCI, respectively (Table 6) (Peters and Timmerhaus 1991).

Due to the high equipment costs for in-house CO_2_ extraction (D1), with €663,000, the resulting annual depreciation on equipment and buildings is higher, compared to solvent extraction from homogenised cyst cells (D2) and zoospores (D3) with €476,000 and €460,000, respectively. The lowest depreciation costs of €360,000 were calculated for an external supercritical CO_2_ extraction (D4). As the fixed charges (B) are derived from the depreciation, local taxes and insurance costs, at €1.1 million, these are also highest for in-house supercritical CO_2_ extraction (D1), followed by €0.78 and €0.76 million for solvent extraction from homogenised cyst cells (D2) and zoospores (D3), as well as €0.59 for supercritical CO_2_ extraction via an external service provider (D4). The plant overhead costs (C) are 50% of the costs of the operating labour, supervisory labour and maintenance and repairs (Peters and Timmerhaus 1991) and are presented in Table 6.

The general expenses (II) are the sum of the administrative costs, distribution and marketing, research and development, and interest payments and are shown in Table 6. An interest rate of 2% and a 100% debt financing of the project were assumed. Due to higher investment costs and therefore higher interest payments, the general expenses for in-house supercritical CO_2_ extraction (D1) are highest at €555,000, followed by €441,000 and €432,000 by solvent extraction from homogenised cyst cells (D2) and flagellated zoospores (D3), as well as €417,000 by external CO_2_ extraction. The total product costs (III) are the sum of the manufacturing costs (I) and general expenses (II), as presented in Table 6 and Fig. 5.

The highest total product costs (III) were determined to be €3.81 million for the conventional process, with an in-house supercritical CO_2_ extraction (D1). Total product costs of €2.98 million and €2.92 million were determined for the alternative process using CPE extraction from homogenised cysts (D2) and germinated zoospores (D3). Total product costs of €3.08 million were calculated for the process in which supercritical CO_2_ extraction is carried out by a service provider (D4). A comparison of the total product costs for scenarios D2 and D3 with D4 shows that the higher direct production costs in D4 (mainly due to the payment of the external service provider for supercritical CO_2_ extraction) are offset by lower costs of the fixed charges (mainly due to lower deprecation for equipment and buildings).

### Economic performance of the four examined downstream scenarios

After determining the TCI and the total product costs (III) in the subsequent “Determination of the total capital investment and total product costs” section, the economic performance of the four downstream scenarios will be discussed.

The return on investment (ROI) and the net present value (NPV) were used as key figures for economic profitability:

5 $${\text{ROI}} = \frac{{{\text{EAT}}}}{{{\text{TCI}}}}.$$

As presented in Eq. 5, the ROI is the quotient of the profit after depreciation, interest, and taxes (EAT) and the TCI (Peters and Timmerhaus 1991).

The discount factor *d*_*n*_ (Eq. 6) is the factor by which the future cash flow must be multiplied to obtain the present value of the cash flow after *n* years if invested at interest *i* (Peters and Timmerhaus 1991):

6 $$d_{n} = \frac{1}{{\left( {1 + i} \right)^{n} }}.$$

The discount factor was defined for yearly payments and annual compounding:

7 $${\text{NPV}} = \sum \limits_{n = 1}^{t} \frac{{{\text{NB}}_{n} }}{{\left( {1 + d} \right)^{n} }}.$$

The NPV of the processes compares the difference between the present value of annual cash flows and the initial required investment (Peters and Timmerhaus 1991). The NPV is calculated according to Eq. 7, where net benefits NB corresponds to the net cash flow at year *n*. The internal rate of return (IRR) was calculated and corresponded to a discount factor at NPV = 0 and gives the interest rate *i* at which the initial investment breaks even with the generated cash flows.

A total of 3241 kg of oleoresin (10 wt% astaxanthin) could be produced in the four downstream scenarios, as shown in Table 9. A sales price of €1200 per kg of oleoresin was assumed, which results in gross revenues of around €3.89 million. The difference between gross revenues and total product cost (III), excluding depreciation and interest payments, is the earnings before interest, taxes, depreciation, and amortisation (EBITDA). The EBITDA is an important economic parameter, as it enables the comparison of the economic performance of different companies regardless of interest payments, type of depreciation, and country-specific taxation. Due to the lowest total production costs, the two alternative downstream processes, using CPE extraction (D2 and D3), showed the highest EBITDA with €1.54 and €1.51 million, respectively. Consequently, a lower EBITDA was reached for the in-house (D1) and external (D2) supercritical CO_2_ extraction from homogenised cyst cells (Table 9).

**Table 9:** Economic key figures for the evaluation of the four downstream scenarios, D1, D2, D3, and D4

		D1	D2	D3	D4
Price of oleoresin (10 wt%_astaxanthin_) per kg	€	1200			
Oleoresin (10 wt%_astaxanthin_) produced per year	kg	3241			
Gross revenue	€	3,889,620	3,889,620	3,889,620	3,889,620
Total product cost	€	3,805,199	2,981,077	2,921,805	3,075,581
Interest	€	172,044	123,399	119,223	93,308
Depreciation	€	662,988	475,530	459,436	359,571
EBITDA	€	919,453	1,507,472	1,546,474	1,266,918
EBIT	€	256,465	1,031,942	1,087,038	907,347
EBT	€	84,421	908,543	967,815	814,039
Profit tax (29%)	€	24,482	263,478	280,666	236,071
EAT	€	59,939	645,066	687,149	577,968
ROI	%	0.62	9.30	10.26	11.03
Operating cash flow	€	722,927	1,120,596	1,146,585	937,539

Due to higher depreciation, interest payments, and paid taxes for processes D2 and D3 compared to D4, the profits after depreciation, interest payments, and taxes (EAT) converge and amount to €0.69 million for process D3, €0.65 million for D2 and €0.58 million for D4. The lowest EAT of €0.06 million was in process D4, due to the highest depreciation and interest payments.

However, the profit itself is not a sufficient parameter for the economic comparison of the processes, as it neglects the TCI to reach the profit (Turton et al. 2012; Peters and Timmerhaus 1991). Therefore, the ROI was calculated as defined in Eq. 5 (Panis and Carreon 2016; Zgheib et al. 2018). The highest ROI of 11% was reached for the downstream process, performing supercritical CO_2_ extraction via a service provider (D4), followed by 10.3% by the solvent extraction from zoospores (D3) and 9.3% from homogenised cyst cells (D2). Due to low EAT and high TCI, with 0.6%, the ROI is lowest for in-house supercritical CO_2_ extraction (D4). However, from costs higher than 65€ per kg biomass for supercritical CO_2_ extraction via an external service provider (D4), the alternative processes of solvent extraction (D2 and D3) would achieve higher ROI than the contracted supercritical CO_2_ extraction. For long-term investments, the need for a NPV adjustment, taking the time value of money into account, is required. As presented in Table 10, the highest NPV was determined for scenario D3 with a value of €2.66 million after an operating time of 10 years. A negative NPV of €3.7 million is reached for the in-house supercritical CO_2_ extraction (D1). The IRR is the discount factor for which the NPV of the project is equal to zero and is the interest rate at which the project can just break even. Typically, rates for IRR are 10% for cost improvement of conventional technologies, 15% for the expansion of conventional technologies, 20% for product development, and 30% for speculative ventures (Van Dael et al. 2015). As shown in Table 10, the highest IRR can be expected for the external supercritical CO_2_ extraction, followed by the new downstream scenarios of solvent extraction from homogenised cyst cells and flagellated zoospores.

**Table 10:** Total present value for an interest rate of 2%, NPV after 10 years and IRR of the four downstream D1, D2, D3, and D4

		D1	D2	D3	D4
TCI		9,665,420	6,932,555	6,697,930	5,242,042
Total present value of discounted cash flows	€	5,900,698	9,146,567	9,358,698	7,652,416
NPV	€	− 3,764,721	2,214,012	2,660,767	2,410,374
IRR	%	<0	8.25	9.65	10.75

## Conclusion

In this study, an alternative downstream process for the extraction of astaxanthin from *H. pluvialis* was developed, replacing the drying of the biomass and supercritical CO_2_ extraction with CPE extraction from homogenised cyst cells or germinated zoospores. Using a CPE unit with a column volume of 244 mL, 3.5 g oleoresin could be extracted from 7.58 g homogenised *H. pluvialis* biomass within 32 min. A scale-up to an industrial 5-L CPE column showed that up to 2,947 kg of biomass could be processed within 330 days (24 h a day) of operation. For the techno-economic study, annual algal production of 8910 kg biomass with 5% astaxanthin was assumed, resulting in daily production of 9.83 kg oleoresin. Lower direct production costs were determined for the two alternative extraction processes using CPE compared to supercritical CO_2_ extraction. Also the total product costs are lower for the two new processes using CPE extraction compared to the supercritical CO_2_ extraction processes. After 10 years of operation, the NPV is highest for the CPE extraction from germinated zoospores. It must be noted that the results of the economic study will vary, depending on the individual situation of the *H. pluvialis* companies (financing, taxes, labour and electricity costs, depreciation, and interest rate). However, especially for small-size companies, the CPE extraction described represents an interesting alternative, as extraction can be performed in-house regularly, and the storage of biomass for shipment to a service provider for supercritical CO_2_ is no longer required.

## Data Availability

The data supporting the conclusions of this article are included in the main manuscript.

## Abbreviations

BBM: Bold modified basal medium
CPE: Centrifugal partition extractor
*d*_*n*_: Discount factor
EAT: Earnings after tax
EBT: Earnings before tax
EBIT: Earnings before interest and taxes
EBITDA: Earnings before interest, taxes, depreciation and amortisation
FCI: Fixed-capital investments
*H. pluvialis*: *Haematococcus pluvialis*
IRR: Internal rate of return
NB: Net benefits
NPV: Net present value
ROI: Return on investment
TCI: Total capital investments
TDIPC: Total direct and indirect planned costs
TEC: Total equipment costs
